# Down-regulation of HMGB1 expression by shRNA constructs inhibits the bioactivity of urothelial carcinoma cell lines via the NF-κB pathway

**DOI:** 10.1038/srep12807

**Published:** 2015-08-04

**Authors:** Zhichao Huang, Zhaohui Zhong, Lei Zhang, Xinjun Wang, Ran Xu, Liang Zhu, Zijian Wang, Shanbiao Hu, Xiaokun Zhao

**Affiliations:** 1 Department of Urology, The Second Xiangya Hospital, Central South University, Changsha 410011, Hunan, China; 2 Department of Urology, Zhongshan Hospital, Xiamen University, Xiamen 361004, Fujian, China

## Abstract

The high mobility group box 1 (HMGB1), which is a highly conserved and evolutionarily non-histone nuclear protein, has been shown to associate with a variety of biological important processes, such as transcription, DNA repair, differentiation, and extracellular signalling. High HMGB1 expression has been reported in many cancers, such as prostate, kidney, ovarian, and gastric cancer. However, there have been few studies of the function of HMGB1 in the malignant biological behaviour of bladder urothelial carcinoma (BUC), and the potential mechanism of HMGB1 in the pathogenesis of BUC remains unclear. Thus, in this study, we constructed plasmid vectors that are capable of synthesizing specific shRNAs targeting HMGB1 and transfected them into BUC cells to persistently suppress the endogenous gene expression of HMGB1. The expression of HMGB1, the bioactivity of BUC cells, including proliferation, apoptosis, cell cycle distribution, migration and invasion, and the effects of HMGB1 knockdown on downstream signalling pathways were investigated. Our data suggest that HMGB1 promotes the malignant biological behaviour of BUC, and that this effect may be partially mediated by the NF-κB signalling pathway. HMGB1 may serve as a potential therapeutic target for BUC in the future.

Bladder carcinoma (BC) is the most common malignancy of the urinary tract and the seventh most common cancer in men and the 17^th^ in women worldwide^1^. In the United States, it has been estimated that nearly 74,690 new cases of BC were diagnosed in 2014, with approximately 15,580 deaths^2^. Despite the development of surgical techniques and instruments, patients with non-muscle invasive BC still experience a high risk of recurrence, and 1/3 of these patients will progress to muscle-invasive BC^3^,^4^. The clinical outcome of muscle-invasive BC is also unfavourable, with an overall survival rate of 48% to 67% within 5 years^5^. Bladder urothelial carcinomas (BUCs) represent nearly 90% of BCs that arise from an epithelial origin. Therefore, it is essential to study the mechanism of BUC development and progression and to identify more effective treatment strategies for this malignancy.

High mobility group box 1 (HMGB1), a nuclear non-histone protein, has been associated with a variety of biological important processes, such as transcription, DNA repair, V(D)J recombination, differentiation, and extracellular signalling^6^,^7^,^8^. Acting as a chromatin-binding factor, HMGB1 exerts its key functions within the nucleus by binding the minor groove of DNA and facilitating the assembly of site-specific DNA-binding proteins, which regulate the transcription of a number of genes^9^,^10^,^11^,^12^. In addition to its nuclear role, HMGB1 can be actively secreted by inflammatory cells and passively released from necrotic cells into the local microenvironment, acting as an extracellular signalling molecule that binds individual surface receptors, including the receptor for advanced glycation end products (RAGE) and Toll-like receptors (TLRs) –2, –4 and –9 during inflammation, cell migration, cell differentiation, and cancer metastasis^13^,^14^. The overexpression of HMGB1 has been confirmed in a variety of cancers, such as prostate cancer^15^, renal cell carcinoma^16^, bladder cancer^17^, hepatocellular carcinoma^18^, gastric cancer^19^, colorectal cancer^20^, and lung cancer^21^. Furthermore, overexpression of HMGB1 is associated with all the hallmarks of cancer, including limitless replicative potential, evasion of apoptosis, angiogenesis, inflammatory microenvironment, and tissue invasion and metastasis, indicating that HMGB1 might be a new potential therapeutic target for the treatment of human malignancies^22^.

There have been few studies of the function of HMGB1 in the malignant biological behaviour of BUC. Thus, in this study, we constructed HMGB1 shRNA plasmid vectors, which can be processed to generate small interfering RNAs (siRNAs), and transfected them into BUC cells to persistently suppress the endogenous gene expression of HMGB1. The expression of HMGB1, the bioactivity of BUC cells, including proliferation, apoptosis, cell cycle distribution, migration and invasion, and the effects of HMGB1 knockdown on downstream signalling pathways were investigated to obtain further insights into the role of HMGB1 in the pathogenesis of BUC and the probable mechanism of this role.

## Results

### HMGB1 expression is up-regulated in BUC tissues and cell lines

**Figure 1:**
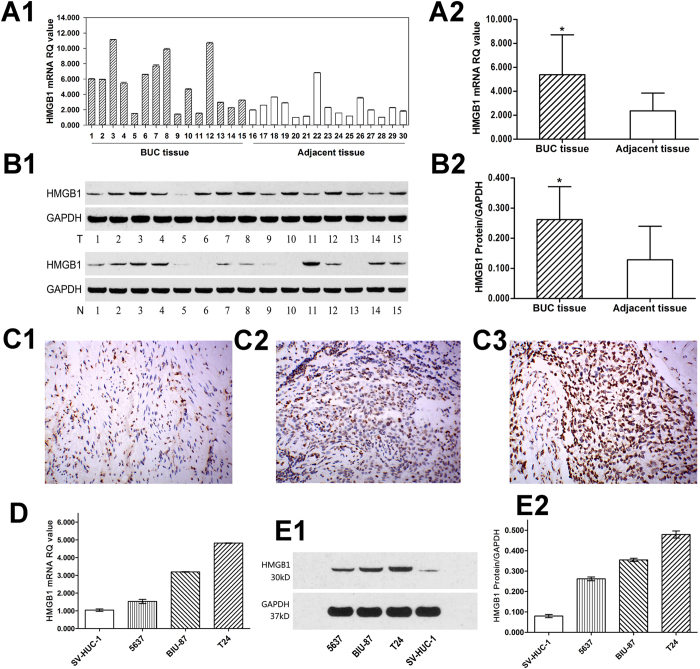
HMGB1 expression is up-regulated in BUC tissues and cell lines. Figure 1A1 and A2: qRT-PCR was performed to evaluate the levels of HMGB1 mRNA in 15 paired fresh BUC and adjacent non-tumour tissues. *P  < 0.05, compared with adjacent non-tumour tissue. Figure 1B1 and B2: The overexpression of HMGB1 protein was detected by WB in 15 BUC and paired adjacent non-tumour tissues (T: BUC tissue; N: adjacent non-tumour tissue). *P  < 0.05, compared with adjacent non-tumour tissue. Figure 1C1, C2, and C3: The expression of HMGB1 in BUC and adjacent non-tumour tissues was examined by IHC (×200). HMGB1 staining was much weaker in the majority of adjacent non-tumour tissues (Fig. 1C1). Among BUC samples, low expression of HMGB1 was detected only in 13/30 (43.3%) of BUC tissues (Fig. 1C2), while high expression of HMGB1 was apparent in 17/30 (56.7%) of BUC tissues (Fig. 1C3). Figure 1D, E1, and E2: qRT-PCR and WB were performed to measure the levels of HMGB1 mRNA and protein in different BUC cell lines. Pairwise significant differences were detected among them, with the HMGB1 expression levels all significantly higher than in the SV-HUC-1 cell line (all *P* < 0.001). Furthermore, among these BUC cells, the expression level of HMGB1 was highest in T24 cells and the lowest in 5637 cells. The display of cropped gels is used to improve the clarity and conciseness of the presentation, and all the cropped gels have been run under the same experimental conditions.

Fifteen BUC tissues and paired adjacent non-tumour tissues were used to detect the expression of HMGB1. Quantitative real-time PCR (qRT-PCR) showed that the HMGB1 mRNA level was significantly higher in BUC tissues compared with paired adjacent non-tumour tissues (5.390 ± 3.329 vs. 2.373 ± 1.485, *P* < 0.05, Fig. 1A1, A2). Similarly, western blotting (WB) analyses showed that the expression of HMGB1 protein was higher in BUC tissues compared with paired adjacent non-tumour tissues (0.262 ± 0.109 vs. 0.129 ± 0.111, *P  *< 0.05, Fig. 1B1, B2). Next, the expression and subcellular localization of HMGB1 protein were detected by immunohistochemistry (IHC) in a series of 30 BUC tissues and paired adjacent non-tumour tissues. According to the criteria described below, high expression of HMGB1 was observed in 56.7% (17/30) of BUC tissues and only 10.0% (3/30) of adjacent non-tumour tissues (*P* < 0.05, Fig. 1C1, C2 and C3). Additionally, expression of HMGB1 in 5637, BIU-87, T24 and SV-HUC-1 cells was detected by qRT-PCR and WB. As shown in Fig. 1D, E1 and E2, HMGB1 mRNA and protein were highly expressed in BUC cell lines compared to the SV-HUC-1 normal urothelial cell line (all *P* < 0.001). Furthermore, among these BUC cells, the expression level of HMGB1 was highest in T24 cells and lowest in 5637 cells.

### RNA interference (RNAi) effectively inhibits HMGB1 expression in BUC cells

**Figure 2:**
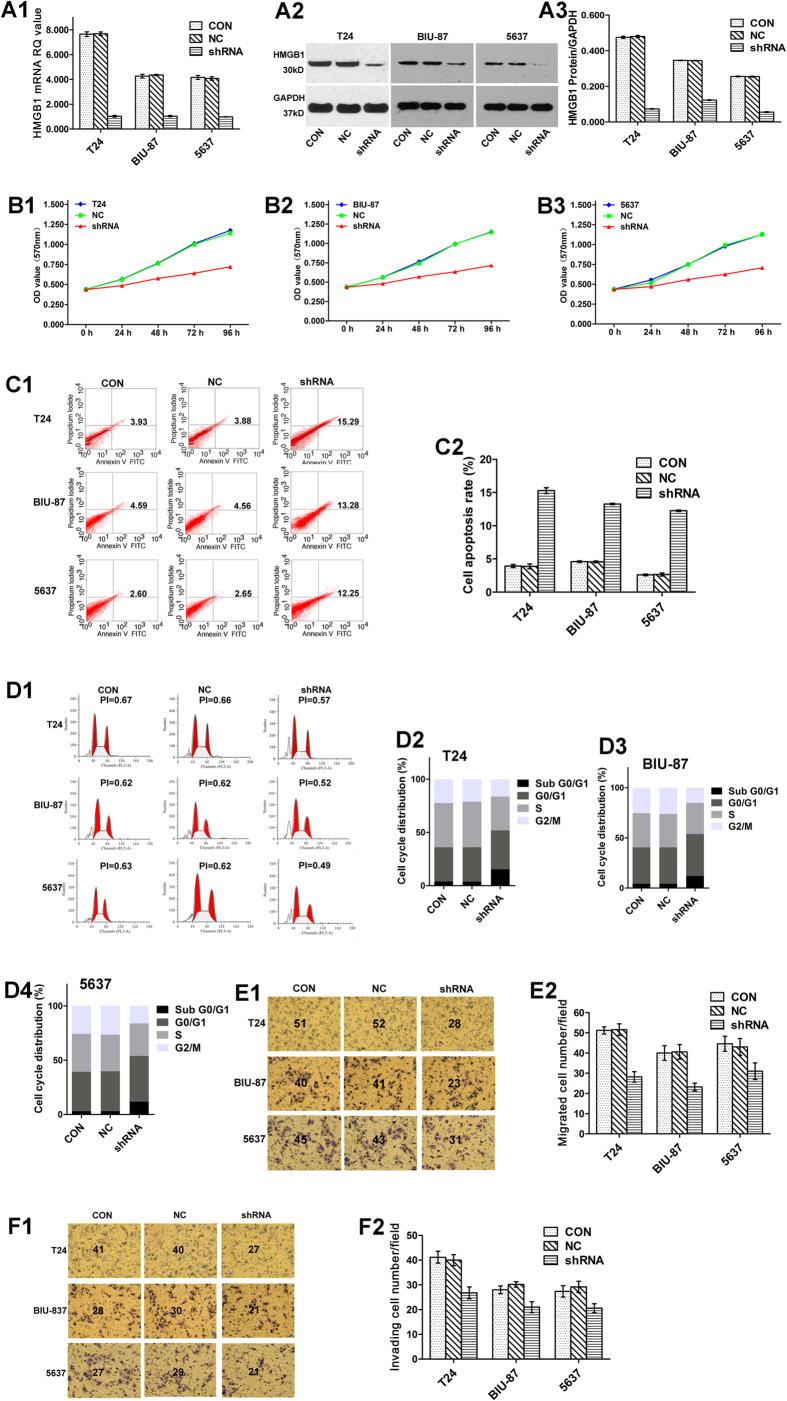
Knockdown of HMGB1 expression inhibits the biological behaviour of BUC cell lines. Figure 2A1, A2 and A3: HMGB1 expression of BUC cell lines was detected in groups after different transfections, which resulted in the differential expression of HMGB1 among the groups in each BUC cell line. The results indicated that the HMGB1 shRNA plasmids significantly inhibited the expression of HMGB1 compared with the CON and NC groups (all *P* < 0.001). No significant differences were found between CON and NC in three BUC cell lines (all *P* > 0.05). Figure 2B1, B2 and B3: Cell proliferation of BUC cell lines evaluated by MTT assay showed that knockdown of HMGB1 expression (shRNA group) resulted in lower cell proliferation than in the CON and NC groups (all *P* < 0.05). Figure 2C1 and C2: Cell apoptosis assays for BUC cell lines indicated that knockdown of HMGB1 expression induced cell apoptosis compared with the CON and NC groups (all *P* < 0.05). Figure 2D1, D2, D3 and D4: Cell cycle phase assays for BUC cell lines demonstrated that knockdown of HMGB1 expression led to an increase in the percentage of cells in G0/G1 phase, with a significantly lower proliferation index (PI) than the other groups (all *P* < 0.05). Figure 2E1, E2, F1 and F2: Cell migration and invasion assays confirmed that knockdown of HMGB1 expression reduced the migration and invasive ability of BUC cell lines *in vitro* compared with the CON and NC groups (all *P* < 0.001).

For the following experiments, three BUC cell lines (T24, BIU-87 and 5637) were divided into three groups: the CON group (non-transfected cells), the NC group (shNC-transfected cells), and the shRNA group (HMGB1 shRNA-transfected cells). WB and qRT-PCR were performed to measure HMGB1 expression after RNAi (Fig. 2A1, A2 and A3). The results showed different expression levels of HMGB1 among the three groups of each cell line. HMGB1 shRNA plasmids significantly inhibited the expression of HMGB1 mRNA and protein compared with the CON and NC groups (all *P* < 0.001). There were no significant differences in the expression of HMGB1 mRNA and protein between the CON and NC groups (all *P* > 0.05). The results of qRT-PCR and WB demonstrated that the RNAi was effective.

### Knockdown of HMGB1 expression inhibits BUC cell proliferation

Following knockdown of HMGB1 gene expression with shRNA, the proliferation of transfected BUC cells was measured using the MTT assay at 24 h, 48 h, 72 h and 96 h post-transfection (Fig. 2B1, B2 and B3). The cell proliferation in the shRNA groups was significantly reduced 24 h after transfection compared with both the CON and NC groups (all *P* < 0.05, Supplementary Table S1). There were no significant differences in cell proliferation between the CON and NC groups in BUC cells (all *P* > 0.05, Supplementary Table S1).

### Knockdown of HMGB1 expression enhances BUC cell apoptosis

As demonstrated in Fig. 2C1 and C2, early and late apoptosis were higher in the cells transfected with HMGB1 shRNA plasmids (shRNA group) than in the CON and NC groups (all *P* < 0.05). No significant differences in cell apoptosis between the CON and NC groups were observed in the BUC cells (all *P* > 0.05).

### Knockdown of HMGB1 expression induces BUC cell cycle arrest

To investigate the mechanism by which the proliferation of BUC cells was suppressed by the down-regulation of HMGB1 expression, flow cytometry was used to detect the specific phase of the cell cycle. The data in Fig. 2D1, D2 D3 and D4 demonstrate that knocking down HMGB1 expression (shRNA group) induced BUC cell arrest in the G0/G1 phase, with a proliferation index (PI) that was significantly lower than the CON and NC groups (all *P* < 0.05). No significant differences were found between the CON and NC groups in the cell cycle assay (all *P* > 0.05).

### Knockdown of HMGB1 expression inhibits BUC cell migration and invasion

As shown in Fig. 2E1, E2, F1 and F2, the number of migrating and invading BUC cells in the shRNA group were significantly lower compared with the CON and NC groups. In addition, the BUC cells in which HMGB1 had been knocked down had lower migration and invasive abilities (all *P* < 0.001), while no significant differences between cell migratory and invasive ability were found for the CON and NC groups (all *P* > 0.05).

### Effects of HMGB1 down-regulation on NF-κB/p65, IκBα and VEGF-C in T24 cells

**Figure 3:**
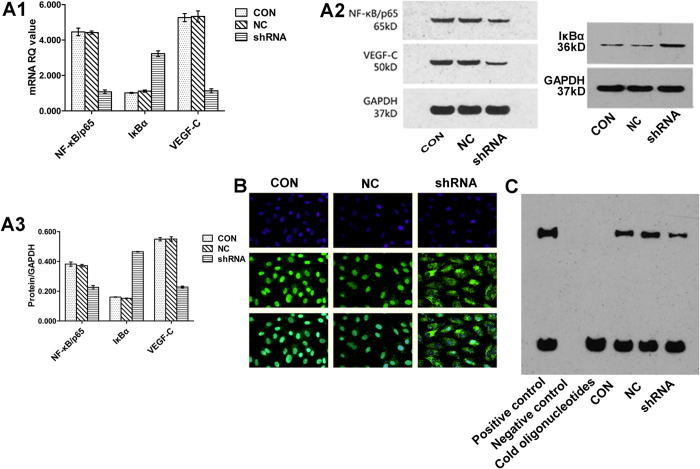
Effects of HMGB1 down-regulation on NF-κB/p65, IκBα and VEGF-C in T24 cells. Figure 3A1, A2 and A3: The expression of NF-κB/p65 and VEGF-C in the shRNA group was lower than in the other two groups transfected with shNC plasmids or the untransfected controls (all *P* < 0.05). On the contrary, the expression of IκBα showed an opposite tendency, while no significant differences in the expression of NF-κB/p65, IκBα and VEGF-C in T24 cells were found between the CON and NC groups (all *P* > 0.05). The display of cropped gels is used to improve the clarity and conciseness of the presentation, and all the cropped gels have been run under the same experimental conditions. Figure 3B: The blue areas indicate nuclei stained using 4, 6-diamidino-2-phenylindole (DAPI), and the green areas indicate the nuclear translocation of NF-κB/p65 in T24 cells transfected with shNC plasmids or untransfected and cytoplasmic localization of NF-κB/p65 in cells transfected with shRNA plasmids. The results showed that knockdown of HMGB1 expression inhibited the translocation of NF-κB/p65 from the cytoplasm to the nucleus. Figure 3C: EMSA revealed that the DNA-binding activity of NF-κB/p65 in T24 cells was decreased by HMGB1 knockdown.

To further understand the probable mechanism of HMGB1 shRNA-mediated biological changes, the expression levels of NF-κB/p65, IκBα and VEGF-C were detected by WB and qRT-PCR. In addition, the nucleic localization and DNA-binding activity of NF-κB/p65 were determined by immunofluorescence and electrophoretic mobility shift assay (EMSA), respectively. As shown in Fig. 3A1, A2 and A3, the protein and mRNA levels of NF-κB/p65 and VEGF-C were significantly lower in cells transfected with HMGB1 shRNA plasmids than in those transfected with shNC plasmids or untransfected (all *P* < 0.05). On the contrary, IκBα expression showed the opposite tendency. There were no significant differences in NF-κB/p65, IκBα and VEGF-C expression between the CON and NC groups (all *P* > 0.05). At 72 h post-transfection, the NF-κB/p65 proteins were stained green and the nuclei of the T24 cells were stained blue. As shown in Fig. 3B, the knockdown of HMGB1 expression by shRNA-mediated RNAi inhibited the translocation of NF-κB/p65 from the cytoplasm to the nucleus. Furthermore, the EMSA assay suggested that the DNA-binding activity of NF-κB/p65 in T24 cells was decreased by HMGB1 knockdown (Fig. 3C).

## Discussion

In the nucleus, HMGB1 is a DNA-binding protein that serves as a structural component to facilitate the formation of nucleoprotein complexes. Outside the cell, HMGB1 can act as a cytokine, which can be released into the extracellular environment by tumour or inflammatory cells undergoing necrosis, or in response to many distinct triggers, including hypoxia, nutrient deprivation, shortage of essential growth factors, or application of radiation and chemotherapy^23^. Thus, it has been demonstrated that HMGB1 plays an important role in a variety of biological processes, including transcription, DNA repair, differentiation, the activation of endothelial cells, and the initiation of inflammation^24^,^25^. Previous studies have confirmed that HMGB1 is overexpressed in a variety of cancers^15^,^16^,^17^,^18^,^19^,^20^,^21^. Furthermore, HMGB1 has been implicated in many hallmarks of cancer, including apoptosis, angiogenesis, invasion, metastasis and inflammatory microenvironment^22^. In this study, we confirmed that both mRNA and protein levels of HMGB1 were significantly higher in BUC tissues and cell lines than in non-tumour cells. To investigate whether HMGB1 could be used as a potential therapeutic target, we constructed specific HMGB1 shRNA plasmids and transfected them into BUC cells. The results showed that shRNA-mediated RNAi efficiently suppressed HMGB1 expression in BUC, which suggests that plasmid-based shRNA targeting HMGB1 expression in BUC cells *in vitro* can be used as a model system for further functional assays.

Using MTT and flow cytometry assays, we found that knockdown of HMGB1 suppressed the proliferation and induced the apoptosis of BUC cells. However, it also increased the number of cells in the G0/G1 phase and reduced the number of cells in the S phase. These results indicate that HMGB1 is important for tumour growth. Chen RC *et al.*^26^ have reported that anti-HMGB1 neutralizing antibody could reduce the cell viability of HCCLM3 hepatocellular cancer cells, while this effect could be reversed by rhHMGB1, which indicated that HMGB1 might play an important role in cell proliferation. Chen J *et al.*^27^ constructed a lentivirus vector with HMGB1 shRNA to specifically suppress the expression of HMGB1 in ovarian cancer cells. They found that the knockdown of HMGB1 expression could decrease the expression of PCNA and Cyclin D1, which controls cell cycle transit, induces cell cycle G0/G1 arrest, and inhibits cell proliferation. These results indicated that HMGB1 might have an effect on cell proliferation by regulating the expression of some cell cycle proteins. Gnanasekar M *et al.*^28^ demonstrated that knockdown of HMGB1 by shRNA plasmids in LNCaP prostate cancer cells could induce apoptosis via a caspase-3 dependent pathway. Volp K *et al.*^29^ found that HMGB1 overexpression increased NF-κB activity and led to c-IAP2 up-regulation in colon carcinoma, which could inhibit apoptosis via suppressing caspase-3 and caspase-9 activity. Brezniceanu M *et al.*^30^ demonstrated that HMGB1 protected mammalian cells against different death stimuli, including caspase-8, CD 95, ultraviolet radiation, TRAIL, and Bax-induced apoptosis.

Metastasis is considered one of the primary causes of death in patients with malignant tumours. Previous studies have demonstrated that HMGB1 may be involved in cancer progression, including angiogenesis, mobility, inflammatory microenvironment, invasion and metastasis^31^,^32^. In this study, we concluded that the down-regulation of HMGB1 could suppress the migration and invasion of BUC cells, indicating that HMGB1 is involved in the metastasis of BUC. Yan W *et al.*^33^ found that HMGB1 played a pivotal role in hepatocellular carcinoma invasion and metastasis by activating caspase-1, with the subsequent production of multiple mediators, including IL-1β and IL-18. Lin L *et al.*^16^ demonstrated that HMGB1 binding to RAGE initiated the signalling pathway and activated ERK1/2, which promoted gene expression, protein synthesis, and the migration and invasion of renal cell carcinoma cells. In lung cancer, Wang C *et al.*^34^ confirmed that HMGB1 binding to RAGE and TLR-4 was critical for the up-regulation of matrix metalloproteinase (MMP) –2, 9 in 95D cells, which could enhance the invasive potential of 95D cells.

NF-κB complexes are composed of homo- or heterodimers formed from the multi-gene family of p65, p50, p52, c-Rel and Rel B^35^. In most cell types, inactive NF-κB is predominantly sequestered in the cytoplasm bound to its inhibitors (IκBs). Upon activation, IκBs are degraded, thus allowing NF-κB translocation to the nucleus, where it acts as a sequence-specific DNA-binding transcription factor involved in the tumourigenesis of many cancers^35^,^36^. Of the five subunits of NF-κB, p65 has the highest transcriptional activity and has been widely studied^37^. As one of the subunits in the VEGF family, VEGF-C has been demonstrated to promote not only angiogenesis but also lymphangiogenesis in cancer progression by binding to its receptors, which makes it more important in tumour progression^38^. Furthermore, the VEGF family is a downstream target gene of NF-κB activation^39^,^40^. In this study, we demonstrated that knockdown of HMGB1 expression significantly inhibited the expression levels of NF-κB/p65 and VEGF-C, up-regulated IκBα expression, and suppressed the nuclear translocation and DNA-binding activity of NF-κB/p65, which indicated that HMGB1 might regulate VEGF-C expression in the development of BUC via the NF-κB signalling pathway.

In conclusion, our results reveal that HMGB1 is overexpressed in BUC. The down-regulation of HMGB1 by shRNA plasmids can specifically and effectively inhibit the proliferation, migration, and invasion of cells, and induce apoptosis and G0/G1 arrest in BUC cells. The action of HMGB1 in the progression of BUC may regulate VEGF-C via the NF-κB signalling pathway. Because of its high efficiency and specificity in knockdown gene expression, silencing HMGB1 gene expression by specific shRNA plasmids could be a potential therapeutic strategy against BUC based on the targeting of the extracellular HMGB1 protein^41^. However, *in vivo* experiments are needed to elucidate the effects and risks of using shRNA-mediated RNAi in the HMGB1 gene. Meanwhile, the precise mechanism by which HMGB1 is involved in the progression and development of BUC needs further study.

## Methods

### Ethics statement

Approval for this study was obtained from the Institutional Research Ethics Committee of the Second Xiangya Hospital, Central South University. Prior consent was obtained from all patients for the use of tissue samples. All samples were harvested and made anonymous according to ethical and legal standards.

### Tissue samples

Samples of BUC tissues and matched adjacent non-tumour tissues were obtained from BUC patients at our department. None of the patients had received either radiotherapy or chemotherapy before surgery, and all the diagnoses were confirmed by histological examination.

### Cell lines and cell culture

The 5637, BIU-87, T24 and SV-HUC-1 cell lines were purchased from Xiangya Medical College (Changsha, China). The cells were maintained in DMEM (Invitrogen, Carlsbad, USA) supplemented with 10% foetal bovine serum (FBS) at 37 °C in a humidified atmosphere containing 5% CO_2_.

### Construction of shRNA plasmids and cell transfection

To minimize the off-target effects of RNAi, we constructed three potential sequences targeting the human HMGB1 gene and selected the shRNA sequence (5′-CACCGCGAAGAAACTGGGAGAGATGTTCAAGAGACATCTCTCCCAGTTTCTTCGCTTTTT TG-3′) for the subsequent experiments (Supplementary Table S2 and Figure S1). The selected HMGB1 shRNA sequence and a scrambled sequence (shNC) with no homology to any known human genes were synthesized and ligated into pGPU6/GFP/Neo plasmid vectors. All constructs were identified by sequence analysis.

The cells were seeded at a density of 5 × 10^4^/well in a 6-well plate 24 h before transfection to achieve more than 70% confluence. For transfection, 7.5 μl of each plasmids and 5 μl of Lipofectamine 2000 (Invitrogen, Carlsbad, USA) were added to 250 μl Opti-MEM I (Invitrogen, Carlsbad, USA) and then mixed gently. The cells were transfected with the different mixtures according to the manufacturer’s instructions. The plate was incubated at 37 °C for 48–72 h until the transfection efficiency was more than 80% and was then used in the experiments described below.

### Western blotting

Total protein extracts were prepared with the Total Protein Extraction Kit (ProMab Biotechnologies, Inc., CA, USA), and nuclear protein was extracted using the NucBuster^TM^ Protein Extraction Kit (Merck Biosciences, Germany) in accordance with the manufacturer’s protocols. The protein concentration was confirmed using a BCA Kit (Beyotime, China). Equal amounts of protein were separated by 12% SDS-PAGE and then electrotransferred to polyvinylidene fluoride membranes (Millipore, Bedford, USA). The membranes were blocked with 5% non-fat dried milk in PBS (37 °C overnight), and incubated for 2 h at 37 °C with specific primary antibodies. The specific antibodies were Mouse HMGB1 antibody (Santa Cruz Biotechnology, Santa Cruz, USA; 1:500 dilution), Rabbit NF-κB/p65 antibody (Boster Biological Technology, Wuhan, China; 1:100 dilution), Rabbit IκBα antibody (Abcam, Cambridge, USA; 1:500 dilution), Rabbit VEGF-C antibody (Boster Biological Technology, Wuhan, China; 1:100 dilution), and Mouse GAPDH antibody (Santa Cruz Biotechnology, Santa Cruz, USA; 1:800 dilution). After washing, the membranes were incubated with horseradish peroxidase-conjugated anti-mouse (1:2000 dilution) and anti-rabbit (1:5000 dilution) antibodies for 1 h at room temperature and the blots were detected using Enhanced Chemiluminescence. The film was scanned and the band intensity was analysed with Gel-Pro Analyzer Software 4.0 (Media Cybernetics, Bethesda, MD) to calculate the integral optical density (IOD). The relative levels of target protein were evaluated using the ratio IOD/IOD GAPDH.

### Quantitative real-time PCR

Total RNA was isolated from tissue samples and BUC cell lines using TRizol reagent (Invitrogen, Carlsbad, USA). The quality of the extracted RNA was assessed by the OD260/OD280 ratio. RNA was reverse transcribed to cDNA using the RevertAid^TM^ H Minus First Strand cDNA Synthesis Kit (Fermentas, Vilnius, Lithuania) according to the manufacturer’s protocols. The qRT-PCR assay was carried out using an ABI PRISM 7900HT Sequence Detection System (Applied Biosystems, Foster City, USA). Each well (25 μl reaction volume) contained 12.5 μl of SYBR Green PCR Master Mix (Applied Biosystems, Foster City, USA), 1 μl of template, 1 μl of each primer, and 10.5 μl ddH_2_O. The PCR reaction conditions included an initial denaturation for 5 min at 95 °C followed by 40 cycles each of denaturation for 20 sec at 94 °C, annealing for 20 sec at 61 °C, extension for 20 sec at 72 °C, and a final extension for 5 min at 72 °C. All experiments were repeated in triplicate. The specificity of amplified products was determined by melting curve analysis. The relative expression levels of mRNA were calculated with the 2^–ΔΔCt^ method^42^ and expressed as the RQ value normalized to β-actin. The primers used in the study were as follows: HMGB1 forward (5′-CTGTCCATTGGTGATGTTGC-3′) and reverse (5′-CTTCCTCCTCCTCCTCATCC -3′); NF-κB/p65 forward (5′-GCGAGAGGAGCACAGATACC-3′) and reverse (5′- CTGATAGCCTGCTCCAGGTC-3′); IκBα forward (5′-ACCTGGTGTCACTCCTGTTGA-3′) and reverse (5′-CTGCTGCTGTATCCGGGTG-3′); VEGF-C forward (5′-GGAAAGAAGTTCCACCACCA-3′) and reverse (5′-TTTGTTAGCATGGACCCACA-3′); and β-actin forward (5′-CATTAAGGAGAAGCTGTGCT-3′) and reverse (5′-GTTGAAGGTAGTTTCGTGGA-3′).

### Cell proliferation assay (MTT)

The cells were seeded in 96-well plates at a density of 1 × 10^4^/well and then transfected with shRNA plasmids and incubated at 37 °C in a humidified atmosphere. At 0 h, 24 h, 48 h, 72 h and 96 h post-transfection, 50 μl of MTT solution (5 mg/ml) was added to each well and incubated for 4 h at 37 °C until the formazan crystals formed. Then, the medium was removed, and 150 μl DMSO was added to dissolve the formazan crystals. The OD (570 nm) was measured using a microplate reader (Bio-Rad, Hercules, CA, USA). All experiments were performed in triplicate. The cell growth curves were calculated as the mean OD values of each group at 5 different time points.

### Cell apoptosis assay

Annexin V-FITC/propidium iodide was used to evaluate cell apoptosis. At 72 h post-transfection, the cells were trypsinized, washed, collected and re-suspended in 200 μl binding buffer and 5 μl Annexin V-FITC for 15 min in the dark at a concentration of 1 × 10^6^ cell/ml. Then, 10 μl propidium iodide and 300 μl binding buffer were added to each sample. The cells were evaluated by flow cytometry (BD Biosciences, USA) and the results were analysed as previously described^43^.

### Cell cycle assay

Seventy-two hours after transfection, the cells were collected, washed and centrifuged at 1000 rpm, and then fixed with 75% cold ethanol at 4 °C overnight. After staining in 10 μg/ml propidium iodide and 10 mg/ml RNase A at room temperature for 30 min in the dark, the percentage of cells in each cell cycle phase was determined with Cell Modifit (Verity Software House, Topsham, USA) by a flow cytometer.

### Cell migration and invasion assays *in vitro*

Cell invasion assays were performed in a 6-well transwell chamber (Corning, Cambridge, USA; pore size 8 μm) precoated with Matrigel (BD Biotechnology, USA). After 72 h of transfection, the cells were trypsinized, collected, washed and suspended in a serum-free medium containing 5% BSA at a cell density of 5 × 10^4^ /ml. Then, the lower chamber was filled with 10% FBS and a 2 ml cell suspension was added to the upper chamber. After incubation with 5% CO_2_ for 24 h at 37 °C, cells on the lower surface were fixed with 95% ethanol, stained with hematoxylin-eosin and counted. Five fields were randomly selected to calculate the invading cells (×400). Similarly, the migration assays were performed as described above but without Matrigel.

### Immunofluorescence

The cells were fixed in 4% formaldehyde and incubated in 0.3% Triton X-100 (Sigma, USA) for 1 h at room temperature. After incubation with a specific primary antibody for NF-κB/p65 at 4 °C overnight, the cells were treated with anti-rabbit IgG (H + L) antibody labelled with Alexa 488 (Molecular Probes, USA; 1:200 dilution) for 1 h at room temperature in the dark. Then, the cells were washed three times in PBS after each treatment described above. A confocal microscope (Olympus, Japan) was used to observe the cells.

### Electrophoretic mobility shift assay

The nuclear extracts were prepared as previously described. After determining the concentrations of protein, the biotin end-labelled probe (sense sequence: 5′-biotin- AGTTGAGGGGACTTTCCCAGGC-3′) corresponding to the consensus of NF-κB/p65 was developed. EMSA was performed with a non-radioactive EMSA Kit (Viagene, USA) according to the manufacturer’s protocol. The biotin-labelled duplex DNA (0.5 μl) was then incubated with 3.0 μl nuclear protein extracts in a binding buffer. The reaction mixtures were separated by electrophoresis on 6.5% polyacrylamide gel in 0.5 × TBE, transferred to a nylon membrane, UV cross-linked, and probed with streptavidin-HRP conjugate.

### Immunohistochemistry

The IHC assay was performed as previously described^44^. Formalin-fixed and paraffin-embedded tissue sections (3 μm) were dewaxed in xylene, rehydrated with ethanol in a descending concentration, and immersed in 3% hydrogen peroxide for 10 min to block endogenous peroxidase activity. Antigen retrieval was carried out by submerging sections into 0.01 mol/L citrate buffer (PH 6.0) in a microwave oven for 20 min. After incubation with 10% normal goat serum at room temperature for 15 min, the sections were incubated with rabbit polyclonal HMGB1 antibody (Abcam, Cambridge, USA; 1:200 dilution) overnight at 4 °C. Then, the horseradish peroxidase-conjugated secondary antibody was used to incubate the sections at room temperature for 30 min. Finally, the sections were stained with 3, 3-diaminobenzidine (DAB), counterstained with hematoxylin, dehydrated and mounted. The negative controls included incubation in PBS without primary antibody.

Each section was evaluated and scored according to the staining intensity and extent as described previously^45^. We evaluated the intensity of staining and grouped them into the following four categories: 0 (no staining), 1 (weak staining), 2 (moderate staining), and 3 (intense staining). Meanwhile, the extent of staining was ranked into the following five categories according to the percentage of positive staining areas: 0 (0%), 1 (1–25%), 2 (26–50%), 3 (51–75%), and 4 (76–100%). The index was obtained by multiplying the intensity and extent of the scores (0–7). The sections with an index of ≥3 were defined as HMGB1 high expression and <3 were classified as low expression.

### Statistical analysis

All statistical analyses were performed using SPSS 17.0 (SPSS Inc., Chicago, USA). The quantitative data are shown as the mean ± standard deviation (SD). Comparisons between two groups were performed using the student’s t-test for continuous variables and the Fisher’s exact test for categorical variables. One-way ANOVA was used to assess the significant differences among multiple groups under various treatments. For all tests, *P* values of <0.05 were considered statistically significant.

## Additional Information

**How to cite this article**: Huang, Z. *et al.* Down-regulation of HMGB1 expression by shRNA constructs inhibits the bioactivity of urothelial carcinoma cell lines via the NF-κB pathway. *Sci. Rep.* **5**, 12807; doi: 10.1038/srep12807 (2015).

## Supplementary Material

Supplementary Information
