# Supplementary material for: Down-regulation of HMGB1 expression by shRNA constructs inhibits the bioactivity of urothelial carcinoma cell lines via the NF-κB pathway
Source: Sci Rep. 2015 Aug 4;5:12807. doi: 10.1038/srep12807 (PMC4523846; doi:10.1038/srep12807)

Down-regulation of HMGB1 expression by shRNA constructs inhibits the bioactivity of urothelial carcinoma cell lines via the NF-κB pathway

Zhichao Huang[1] Zhaohui Zhong[1] Lei Zhang[1] Xinjun Wang[2] Ran Xu[1] Liang Zhu[1] Zijian Wang[1] Shanbiao Hu[1] Xiaokun Zhao[1,*]

[1]Department of Urology, The Second Xiangya Hospital, Central South University, Changsha 410011, Hunan, China.

[2]Department of Urology, Zhongshan Hospital, Xiamen University, Xiamen 361004, Fujian, China.

*Correspondence: zhao_xiaokun@yahoo.com

Supplementary Table S1：The 570nm OD values among three groups of BUC cells in MTT assay at 0, 24, 48, 72 and 96h.

	OD value (570nm)		
	CON group	NC group	shRNA group
T24			
0h	0.440±0.007	0.439±0.017#	0.436±0.010*
24h	0.566±0.008	0.563±0.003#	0.486±0.007*
48h	0.767±0.012	0.763±0.012#	0.577±0.012*
72h	1.011±0.021	1.000±0.043#	0.641±0.011*
96h	1.175±0.037	1.141±0.071#	0.722±0.018*
BIU-87			
0h	0.438±0.006	0.436±0.010#	0.436±0.006*
24h	0.563±0.006	0.561±0.004#	0.481±0.008*
48h	0.764±0.010	0.760±0.006#	0.569±0.009*
72h	0.991±0.020	0.993±0.018#	0.634±0.010*
96h	1.149±0.012	1.144±0.022#	0.716±0.008*
5637			
0h	0.438±0.010	0.435±0.006#	0.435±0.006*
24h	0.554±0.006	0.555±0.006#	0.471±0.008*
48h	0.750±0.009	0.749±0.007#	0.559±0.011*
72h	0.979±0.013	0.994±0.009#	0.625±0.007*
96h	1.130±0.019	1.128±0.031#	0.709±0.011*

* $P < 0.05$, compared with the CON and NC groups; # $P > 0.05$, compared with the CON groups.

Supplementary Table S2: The sequences of designed shNC and shRNA targeting human HMGB1 gene.

	sequences
shNC	5’-CACCGTTCTCCGAACGTGTCACGT**CAAGAGATT**ACGTGACACGTTCGGAGAATTTTTG-3’
shRNA-321	5’-CACCGTCTGCTAAAGAGAAAGGAAA**TTCAAGAGA**TTTCCTTTCTCTTTAGCAGACTTTTTTG-3’
shRNA-539	5’-CACCGCGAAGAAACTGGGAGAGATG**TTCAAGAGA**CATCTCTCCCAGTTTCTTCGCTTTTTTG-3’
shRNA-2508	5’-CACCGCAAGTATTCGGTGCTATATA**TTCAAGAGA**TATATAGCACCGAATACTTGCTTTTTTG-3’

The underlines indicate the target sequences for HMGB1, and the bold letters show the loop sequences.

Supplementary figure legend:

Supplementary Figure S1: The interference efficiency of three potential shRNA sequences. T24 cells were either not transfected or transfected with shNC or HMGB1-shRNA plasmids (shRNA-321, shRNA-539 and shRNA-2508). Figure S1A, S1B1 and S1B2: Western blotting and quantitative real-time PCR demonstrated that

all three shRNA plasmids significantly inhibited the expression of HMGB1 protein and mRNA compared with the CON and NC group (all $P < 0.001$). The construct shRNA-539, which induced the best interference effect, was selected to regulate HMGB1 expression in the following study. The display of cropped gels in figures is used to improve the clarity and conciseness of the presentation. The cropped gels were all run under the same experimental conditions.

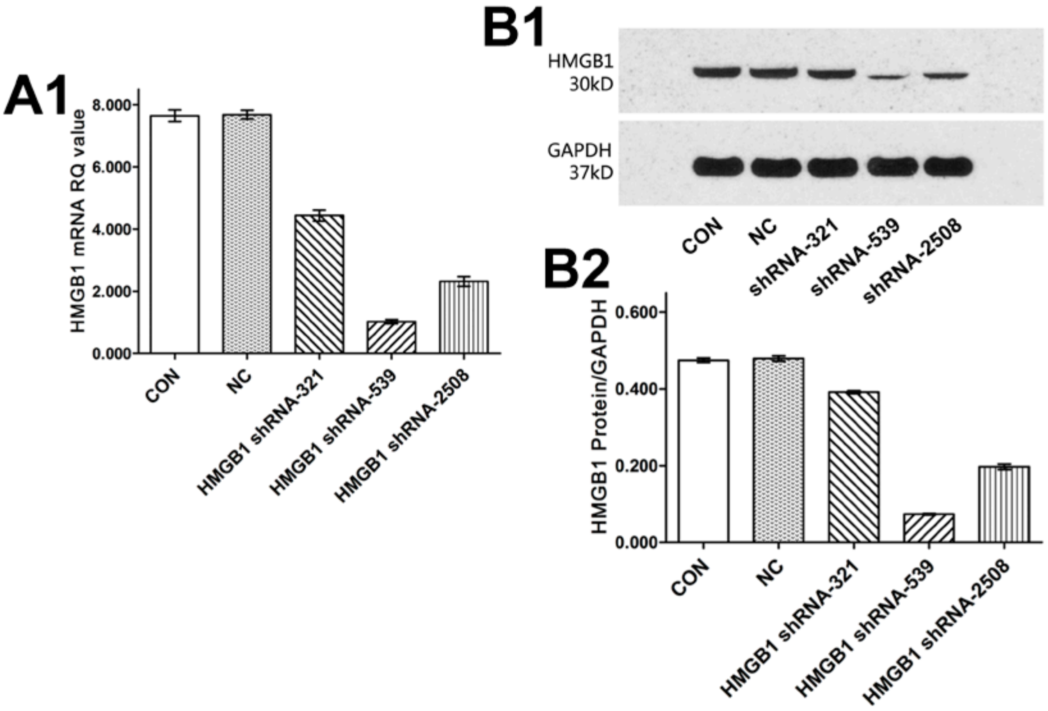

Supplement: Supplementary Information [file srep12807-s1.pdf]
